# The value of brachytherapy in the age of advanced external beam radiotherapy: a review of the literature in terms of dosimetry

**DOI:** 10.1007/s00066-021-01867-1

**Published:** 2021-11-01

**Authors:** Tibor Major, Georgina Fröhlich, Péter Ágoston, Csaba Polgár, Zoltán Takácsi-Nagy

**Affiliations:** 1 Radiotherapy Centre, National Institute of Oncology, Budapest, Hungary; 2 Department of Oncology, Semmelweis University, Budapest, Hungary; 3 Faculty of Science, Eötvös Loránd University, Budapest, Hungary

**Keywords:** Brachytherapy, External beam therapy, Dosimetry, Comparison, Treatment planning

## Abstract

Brachytherapy (BT) has long been used for successful treatment of various tumour entities, including prostate, breast and gynaecological cancer. However, particularly due to advances in modern external beam techniques such as intensity-modulated radiotherapy (IMRT), volume modulated arc therapy (VMAT) and stereotactic body radiotherapy (SBRT), there are concerns about its future. Based on a comprehensive literature review, this article aims to summarize the role of BT in cancer treatment and highlight its particular dosimetric advantages. The authors conclude that image-guided BT supported by inverse dose planning will successfully compete with high-tech EBRT in the future and continue to serve as a valuable modality for cancer treatment.

Brachytherapy (BT) has a long history of successful use in the treatment of various cancers with excellent clinical outcomes, but its use rate within radiotherapy has varied over time. During the first decade of this century there was a steady increase in prostate, breast and gynaecological BT in Europe [1], but since then its use has declined worldwide, particularly in the USA, due to its invasive nature and the widespread availability of high-tech teletherapy [2, 3]. Recently there have been many concerns about its future, and some experts believe that modern external beam techniques, such as intensity-modulated radiotherapy (IMRT), volume modulated arc therapy (VMAT) and stereotactic body radiotherapy (SBRT) with a conventional or robotic linear accelerator could replace it completely. A highly conformal dose distribution can be achieved by complex modulation of external beams, but this can be inherently realized in BT by placing sealed radioactive sources in or near the tumour with the appropriate geometry. Furthermore, due to the steep dose gradient around the sources, the surrounding normal tissues and organs at risk (OARs) receive relatively low doses. Its outstanding dosimetric characteristics have made BT a clinically proven and accepted effective treatment modality for cancer patients for decades. Although BT has been shown to be a cost-effective treatment option with similar outcomes for patients with localised prostate cancer, a decrease in BT use relative to external beam radiation therapy (EBRT) was observed in the United States in the first decade of the century [4–8]. One reason for this was the increasing use of IMRT over the same period [9]. For intermediate- and high-risk patients, a decrease in BT boost after EBRT was also observed despite having a survival advantage over EBRT [10]. However, in contrast to the general trend, increasing use of the BT boost for prostate cancer has recently been reported in Canada, but with significant geographic variation [11].

However, other opinions have also been expressed in support of BT. Evidence suggests that BT is considered mandatory in the treatment of locally advanced cervical cancer, and concerns have been raised that some physicians are trying to replace it with an EBRT boost [12]. In cervical cancer, a decrease in the use of BT is thought to be a cause of poorer survival when modern EBRT techniques are used [13]. Moreover, according to the National Comprehensive Cancer Network (NCCN) guidelines version 4.2019, “SBRT is not considered an appropriate routine alternative to brachytherapy” and “Brachytherapy is a critical component of definitive therapy for all patients with primary cervical cancer who are not candidates for surgery” [14]. According to an analysis of the National Cancer Data, use of BT in the treatment of cervical cancer in the USA has decreased, while at the same time, use of IMRT and SBRT increased significantly between 2004 and 2011, and the IMRT or SBRT boost resulted in poorer overall survival compared to BT [2]. As the physical dose distribution of intracavitary and interstitial BT for the cervix is optimal, and imaging techniques for 3D planning are becoming increasingly available, this technique must be made widely available worldwide [15].

The aim of the present study is to provide a dosimetric overview of the role of BT in the treatment of different anatomical sites of cancer patients in the age of advanced external beam radiotherapy. Following a thorough literature search, the dosimetric assessment was based on a review of relevant publications and our own experience.

## Materials and methods

Studies comparing the dosimetry of BT and EBRT were collected from the literature and the advantages and disadvantages of BT compared to EBRT were evaluated. Data collection was performed by an online search of the PubMed database and a systematic review of the tables of contents of standard radiation oncology journals. For the online search the following keywords and their various combinations were used: “brachytherapy”, “conformal external beam radiotherapy”, “3D-CRT”, “intensity-modulated radiation therapy”, “IMRT”, “volumetric modulated arc therapy”, “VMAT”, “stereotactic body radiotherapy”, “SBRT”, “CyberKnife”, “dosimetry” and “dosimetric comparison”. Studies from 2000 onwards were accepted for evaluation and the last search was carried out in June 2021.

Only studies with at least five patients were selected for detailed analysis. Articles were grouped and screened by treatment site.

Volume (Vx) and dose (Dx) parameters were recorded and compared. Vx is the percentage of the organ volume receiving at least x% of the prescribed dose (PD), and Dx is the minimum relative or absolute dose of the PD delivered to x% of the organ. Some studies have also compared the conformal index (COIN). The COIN is c_1_ × c_2_, where c_1_ is the ratio of the volume of the PTV receiving at least the PD to the volume of the PTV, and c_2_ is the ratio of the volume of the PTV receiving at least the PD to the volume receiving at least the PD.

## Results

BT has been used for more than a century to successfully treat various cancers, and numerous studies have investigated its dosimetric characteristics compared to new emerging EBRT techniques. Table 1 presents the 53 publications that include comparative data from at least 5 patients. The table indicates the number of patients included in the study and the reference CT image set used for comparison. The CTV–PTV margins used in the EBRT studies are also shown in the table. We found one study for brain and head and neck, two for lung, three for liver, six for skin and eight for breast tumours. Most studies were published for gynaecological and prostate cancer (16 for both).

**Table 1:** Studies about dosimetric comparison between brachytherapy (BT) and external beam radiation therapy (EBRT) with data of at least 5 patients

Site/author	EBRT technique	Patient no.	Reference plan with contours	CTV–PTV margin^a^ (mm)	Comments
*Brain*					
Milickovic [16]	SRS	5	SRS	NR	20 non-coplanar beams for SRS
*Breast*					
Patel [17]	3D-CRT, HT	13	BT	10, 5, 0	Margin: 3D-CRT 10 mm, supine HT 5 mm, prone HT 0 mm
Charagvandi [18]	VMAT	20	VMAT	NR	Single-fraction ablative radiotherapy
Terheyden [19]	3D-CRT	136	Two patient cohorts	NR	Boost was given to 36 patients with BT and to 100 with 3D-CRT
Major [20]	IMRT	34	BT	5	4–5 coplanar beams for IMRT
Shahbazian [21]	3D-CRT	15	BT	5	Deeply seated tumours
Fröhlich [22]	SBRT with CK	25	CK	2	Virtual BT plans on CTs with contours for CK
Herein [23]	SBRT with CK	32	Two patient cohorts	2	Comparison between real treatment plans
Periasamy [24]	VMAT	50	Two patient cohorts	5	Tumour bed boost after whole-breast irradiation
*Gynaecological*					
Georg [25]	IMRT, IMPT	9	BT	3–5	High-tech EBRT vs. high-tech BT
Shwetha [26]	IMRT	10	BT	NR	Pear-shaped dose distribution of BT mimicked by IMRT
Gielda [27]	HT	6	BT	0	3–5 mm CTV–PTV margin for BT and HT
Sharma [28]	IMRT	12	BT	3–5	Interstitial BT vs. IMRT for cervical cancer
Cengiz [29]	SBRT with CK	11	BT	0	BT with a tandem and two ovoids with dosimetry on point A
Neumann [30]	CK	11	CK + real BT patients	0	Emulating BT-like inhomogeneous dose distributions
Khosla [31]	IMRT	15	BT and IMRT	10	Two series of CT scans, one for BT and one for IMRT
Otahal [32]	CK	10	BT	0	MRI-based intracavitary and interstitial BT
Pinzi [33]	IMRT	15	BT	7–10	BT and IMRT for boost only
Wali [34]	VMAT	10	BT	5	Boost with VMAT in replacing BT
Jones [35]	SBRT with HT	15	BT	2	Endometrium, 2 mm CTV–PTV margin in HT plans
Kauffmann [36]	3D-Arc and SBRT	6	BT	5	PTV for EBRT was created from 100% isodose cloud of BT
Cilla [37]	VMAT	8	BT	3	Treatment of vaginal cuff in postoperative endometrial cancer
Aydogan [38]	IMRT	10	BT	0	Postoperative vaginal cuff boost for endometrial cancer
Yildrim [39]	VMAT	8	BT	5	Treatment of vaginal cuff in postoperative endometrial cancer
Pedicini [40]	VMAT	27	BT	2	Treatment of the vaginal vault
*Head and neck*					
Akiyama [41]	VMAT	38	BT	0	Identical PTVs in VMAT and BT plans
*Liver*					
Pennington [42]	SABR	9	SABR	0	Planning goal was equal target volume coverage by PD
Hass [43]	SBRT	85	BT	5–10	5–10 mm margin for tumour motion in SBRT plans
Walter [44]	SBRT	38	BT	6	46 treatment plans, 6 mm CTV–PTV margin for SBRT
*Lung*					
Milickovic [16]	SBRT	5	SBRT	NR	9–10 non-coplanar beams for SBRT
Chan [45]	SBRT	9	BT	3	VMAT-based MLC-tracking SBRT
*Prostate*					
Hsu [46]	3D-CRT	5	BT	10	7‑field conformal EBRT plans
Fuller [47]	SBRT with CK	10	SBRT	3–5	Ideally placed catheters in virtual BT plans
Hermesse [48]	IMRT and HT	10	BT	4–10	The same target coverage for each technique
Hermesse [49]	IMRT	10	BT	4–10 and 0	Additional IMRT plans with 0 mm CTV–PTV margin
Murali [50]	IMRT	10	Two patient cohorts	3–5	Step-and-shoot IMRT with seven coplanar beams
Sudahar [51]	CK	13	Two patient cohorts	NR	HDR equivalent doses to CK doses
Spratt [52]	SBRT	5	BT	NR	PTV_100_ of virtual SBRT was matched to V_100_ of BT
Georg [53]	VMAT, IMPT, IMIT	10	VMAT	5–8	HDR/LDR BT techniques, IMRT for protons and ions
Fukuda [54]	SBRT with CK	6	BT	2	Common CT images and contouring sets
Andrzejewski [55]	VMAT, IMPT	12	VMAT	4–5	Boosting to dominant intraprostatic lesions
Yang [56]	VMAT	10	VMAT	5	HDR and LDR BT plans were created
Eade [57]	SBRT	21	SBRT	7	Escalation of boost dose for the GTV
Sanmamed [58]	VMAT	40	Two patient cohorts	3–5	Integrated VMAT boost vs. HDR-BT boost
Chatzikonstantinou [59]	SBRT with CK	15	SBRT	1–3	Virtual HDR-BT plans
Fröhlich [60]	VMAT with CK	10	CK	3	LDR- and single-fraction HDR-BT
Willigenburg [61]	IMRT with MR-linac	30	BT	1	Focal salvage treatment, 1 mm CTV–PTV margin for IMRT
*Skin*					
Park [62]	Electron and photons	6	NA	5	All plans were virtual for electron, photon, IMRT and VMAT
Boman [63]	VMAT	7	BT	3	4 scalp and 3 lower leg moulds
Sen [64]	Electron	10	BT	0	Electron plans with different energies and bolus thicknesses
Mitra [65]	VMAT	12	BT	NA	PTV for VMAT was created from 100% BT isodose volume
Wills [66]	VMAT	14	BT or VMAT	5	9 scalp and 5 extremities moulds
Buzurovic [67]	Electron	37	BT	NR	EBRT plans with 6 MeV electrons and 1 cm bolus

*SRS* stereotactic radiosurgery, *NR* not reported, *VMAT* volume modulated arc therapy, *IMRT* intensity-modulated radiotherapy, *BT* brachytherapy, *CK* CyberKnife, *SBRT* stereotactic body radiotherapy, *SABR* stereotactic ablative radiotherapy, *HT* helical tomotherapy, *IMPT* intensity-modulated proton beam therapy, *IMIT* intensity-modulated carbon-ion therapy, *MLC* multileaf collimator, *HDR* high-dose-rate, *LDR* low-dose-rate, *PD* prescribed dose, *NA* not applicable

^a^In EBRT plans

Six articles with very few patients were found that examined technical considerations and/or the feasibility of using EBRT as an alternative to BT. Three studies, using only one patient, investigated whether 3D-CRT and IMRT can achieve a brachy-like dose distribution for cervical cancer [68–70]. BT irradiation of the vaginal cuff of patients with endometrial cancer has also been simulated with advanced EBRT using two and four patients, respectively [71, 72]. A treatment planning study compared BT dosimetry with 3D-CRT and IMRT in a patient with squamous cell carcinoma of the scalp [73].

As there are no generally accepted and commonly used dose–volume parameters for evaluation of the plans, different quantifiers have been used in the studies, making comparisons difficult. The most commonly used parameters in the reviewed studies are shown in Table 2.

**Table 2:** The most common dose–volume parameters used for comparison in the studies

Treatment site	CTV/PTV	Organs at risk
Brain	V100, V150, V90, V95 D_min_, CI, COIN	D_max_, D_mean_, V_PD_
Breast	V100, V95, V90, D90, DHI, COIN, CI	D_max_, D_mean_, D_0.01_ _cm3_, D_0.1_ _cm3_, D_0.2_ _cm3,_ D_1cm3_, D_2cm3_ V100, V90, V75, V50, V25, V5
Gynaecological	V100, V90 D_min_, D90, DHI, CI, EUD	D_mean_, D_0.1_ _cm3_, D_1cm3_, D_2cm3_, D_5cm3_, D_20cm3_, D10, D50, D90 V150, V10
Head and neck	V100, V95, V90, D90, D100	D_mean_, D_0.1_ _cm3_, D_1cm3_, D_2cm3,_ D10, D30, D50 V10, V30, V50
Liver	V100, V90, D90, D50, D2 D_min_, COIN, CI	D_mean_, V66, V33, V25
Lung	V100, V150, V90, V95 D_min_, CI, COIN	D_max_, D_mean_, D_1cm3_, D_1.2_ _cm3_, D_5cm3_, D_1000cm3_, D_1500cm3_, V_PD_ V15, V20, V30
Prostate	V100, V150, V95, D95, D90 D50, D10, D2, D_min_, COIN, HI	D_max_, D_mean_, D2, D5, D10, D20, D30, D50, D_0.1_ _cm3,_, D_1cm3_, D_2cm3_, V100, V90, V50, V30, V10
Skin	D_min_, D95, D90, D_0.1_ _cm3_, D_0.5_ _cm3_ D_2cm3_, CI, COIN	D_mean_, D_0.1_ _cm3_, D50

*Vx* percentage of organ volume receiving at least x% of the prescribed dose (PD), *Dx* relative or absolute dose irradiating x% or x cm^3^ of the organ volume, *CI* coverage index, *COIN* conformal index, *CTV *clinical target volume, *PTV* planning target volume

### Brain

In a study by Milickovic et al. [16], treatment plans of five patients with recurrent glioblastoma multiforme treated with stereotactic radiosurgery were re-planned as a simulation of HDR-BT. They used identical structure sets and dose prescriptions. The dose gradient at the target volume surface was steeper with BT, resulting in less intermediate doses. With BT, the target coverage and conformity were slightly better, the maximum dose to the brainstem was lower, but the lenses received higher doses with no statistical difference.

### Breast

BT has been used for partial breast irradiation for decades, initially only in clinical trials, but it is now a standard treatment method in selected patient groups due to its good clinical results [74]. Boost doses following whole-breast irradiation can also be effectively delivered with BT [19, 75]. Charagvandi et al. [18] have dosimetrically compared single-fraction ablative RT with interstitial BT and VMAT in early-stage breast cancer. The PD was 15 Gy to the PTV and 20 Gy integrated boost to the GTV. The CTV was created from the GTV by a 2-cm extension (this corresponds to the PTV in BT), and an additional 3‑mm margin was added around the CTV to obtain the PTV for the VMAT plans. For both treatment techniques, the same preoperative CT scans of 20 patients with identical contours were used. Two partial arcs were used in VMAT planning and BT plans were calculated with inverse planning simulated annealing (IPSA) optimization using a median of 16 catheters. The target coverage for GTV and PTV was excellent for both techniques (100% and 99%, respectively). The median of mean ipsilateral lung doses was smaller with BT (0.8 Gy vs. 1.3 Gy, *p* < 0.05). The same was true for the heart, with values of 0.4 Gy and 0.7 Gy, respectively (*p* < 0.05). The median V_2.8_ _Gy_ was also less with BT (0 Gy vs. 0.3 Gy, *p* < 0.05), and its maximum value was only 0.2 Gy in BT compared to 5.9 Gy in VMAT. There was no difference in doses to skin represented by D_1cm3_, (15.9 Gy vs. 15.2 Gy), but the contralateral breast received less dose with BT (median D_mean_: 0 Gy vs. 0.2 Gy, *p* < 0.05). The authors concluded that both techniques are feasible for this two-dose level, single-fraction ablative radiotherapy for early-stage breast cancer, but the PTV is overdosed with BT. We note that this is a natural consequence of an inhomogeneous dose distribution with hotspots around the sources. A detailed dosimetric comparison of multicatheter interstitial BT (MIBT) and IMRT plans for partial breast irradiation was performed by Major et al. [20]. For 34 patients with early-stage breast cancer treated with interstitial HDR-BT, additional IMRT plans were created using identical CT data and contours. In IMRT plans, the PTV was created from the CTV using a 5-mm isotropic margin. The dose prescription was the same for both techniques, i.e., 7 × 4.3 Gy (total 30.1 Gy). In the BT plans, the mean catheter number was 15 (range 7–28), and geometrical and graphical optimization was used. The IMRT plans were generated with 4–5 fixed-gantry coplanar fields with 6‑MV energy photon beams using a sliding-window technique. Although the V100 for the ipsilateral non-target breast was higher with BT (2.4% vs. 0.4%), the medium isodose lines more closely surrounded the PTV in BT plans, resulting in less dose to the breast (V75: 6.1% vs. 11.7%, *p* < 0.05, V50: 13.7% vs. 25.5%, *p* < 0.05). The ipsilateral lung was better protected with BT, all parameters were significantly smaller (e.g., mean lung dose: 5.1% vs. 7.1%, D_2cm3_: 36.5% vs. 50.2%, *p* < 0.05). The heart dosimetry was generally better for IMRT. The mean heart dose for left-sided lesions (*n* = 21) was 2.0% for IMRT and 4.5% for BT (*p* < 0.05), but the D_2cm3_ was lower with BT (18.3% vs. 19.7%, *p* = 0.0129). For skin the D_1cm3_ was 60.2% for BT and 87.8% for IMRT (*p* < 0.05), and regarding the ribs, all parameters were significantly lower with BT. Dose to contralateral breast and lung was also smaller with BT, with a significant difference for D_0.1_ _cm3_ and D_1cm3_.

Interstitial BT was also compared with 3D-CRT for breast cancer [19, 21, 76]. Using a relatively large patient cohort with 136 patients, Terheyden et al. [19] found significant dose reductions to organs at risk using an HDR-BT boost compared to 3D-CRT. In a study with 15 patients, boost doses to deep-seated tumour beds were calculated for interstitial BT and EBRT (3D-CRT photon or electron) [21]. The target coverage was similar for BT and EBRT, but the conformity was best with BT. The COIN was 0.67 for BT and less than 0.3 for EBRT. The volume of the ipsilateral breast exposed to medium to high doses (>50%) was smaller with BT, and the skin, ribs and lung were better protected with BT in this dose range. The EBRT was more favourable for low doses. The mean heart dose was higher with BT (1.69 Gy vs. 0.33 Gy, biologically equivalent doses, *p* < 0.05). In another study, treatment plans of 30 patients treated with interstitial BT and another 30 with 3D-CRT were compared [76]. Doses to OARs were significantly lower with BT. For example, for ipsilateral breast the mean V100 was 13% vs. 15% (*p* = 0.05) and the V50 was 25% vs. 50% (*p* < 0.05). For ipsilateral lung the V30 was 1% vs. 8% (*p* < 0.05), and for heart the D_max_ was 25% vs. 49% (*p* < 0.05), respectively.

Following the previous study with IMRT, the same group investigated the dosimetric characteristics of stereotactic irradiation with CyberKnife (CK) (Accuray Inc., Sunnyvale, CA, USA) in treatment of the partial breast. First, virtual MIBT plans were created on CT images used for CK treatments [22]. In the more conformal CK plans, the doses to skin, ipsilateral lung and ribs were higher compared to BT: skin D_1cm3_ was 86.1% vs. 46.1% (*p* = 0.0018), ipsilateral lung D_1cm3_ 45.0% vs. 38.4% (*p* = 0.0272) and ribs D_1cm3_ 73.6% vs. 49.0% (*p* = 0.0013), but the heart was slightly better protected with CK (D_1cm3_ 10.5% vs. 11.2%, *p* = 0.0534). Then, in another study, MIBT and CK were dosimetrically compared using two separate patient cohorts [23]: 32 APBI patients received their treatments with MIBT and another 32 with CK. The protocol for target volume definition and the fractionation were identical in the two groups. CK plans were more conformal, but the skin and ribs were better protected with MIBT. The mean D_1cm3_ for skin was 58.4% vs. 79.0% (*p* = 0.0007), and for ribs 43.1% vs. 69.0% (*p* = 0.0004) in the MIBT and CK plans, respectively. The mean doses to the ipsilateral lung were the same (4.9%), but doses to most exposed small volumes were significantly smaller for MIBT. The heart parameters were smaller with CK, but the differences were not significant. Doses to contralateral breast and lung were small with both techniques, but CK resulted in smaller values. We note that in both studies [22, 23], CK treatments were performed with tracking and an MLC-based step-and-shoot IMRT technique, and an isotropic margin of 2 mm around the CTV was used to create the PTV.

Analysis of the pooled data from the three studies [20, 22, 23] shows that the mean V50 values for the non-target breast are 10.0% vs. 15.3%, D_1cm3_ values for the ipsilateral lung are 37.8% vs. 48.2%, D_1cm3_ values for the skin are 54.9% vs. 84.3%, and D_1cm3_ values for the ribs are 45.9% vs. 70.6% in favour of BT.

### Gynaecological cancers

#### Cervix

Gynaecological cancers are the most common treatment sites for BT. For the first time, Schefter et al. [68] simulated how IMRT can mimic the dose distribution of a BT treatment for cervical cancer. For a single case, they contoured the reference isodose curve of a standard BT treatment as the target volume for IMRT and optimized the dose distribution of IMRT on this. Without detailed dosimetric analysis, IMRT was found to be able to reproduce the BT dose distribution to a large extent. In a similar study, the dose distribution of a tandem and ovoids was duplicated by IMRT, resulting in a good agreement in the 100% isodose envelope, with a steeper dose falloff for BT [69]. Using data of 9 patients with locally advanced cervical cancer, Georg et al. [25] compared high-tech EBRT with high-tech BT. For BT treatments, intracavitary applicators and interstitial needles were used. In addition to the BT plans, photon IMRT and intensity-modulated proton therapy (IMPT) plans were also prepared and compared. Due to optimization constraints in the EBRT plans, the D_1cm3_ and D_2cm3_ for rectum, bladder and sigmoid were very similar for all plans, but at the cost of lower D90 of the PTV in EBRT plans compared to BT. The total volumes irradiated with doses higher than 3 Gy, 5 Gy and 7 Gy were about twice as large for EBRT as for BT. From their study, the authors conclude that both IMRT and IMPT appear to be inferior to BT. In another study, for 10 cervical cancer patients, Shwetha et al. [26] generated a pear-shaped dose distribution of HDR-BT using sliding-window IMRT. Both D_1cm3_ and D_1cm3_ for the rectum were significantly lower with BT (77.4% vs. 87.7%, *p* = 0.0021 and 69.1% vs. 81.4%, *p* = 0.0006, respectively), and the bladder also received less dose with BT (D_1cm3_: 81.0% vs. 88.7%, *p* = 0.162). There was also a significant difference in the volume of normal tissue receiving 10% of the PD (33.1% vs. 38.8%, *p* = 0.0006). It was concluded that IMRT may be the treatment of choice for cervical cancer when HDR-BT is not available or when a noninvasive treatment technique is preferred. IMRT was compared to interstitial BT for cervical cancer by Sharma et al. [28]. Twelve patients who were not suitable for intracavitary BT were treated with interstitial BT, with a mean of 18 needles (range 14–26). Then, parallel IMRT plans were prepared and compared with the BT plans. Due to the large number of needles used in BT, the dose distributions were more conformal (conformity index defined as volume covered by the referenced isodose/volume of the PTV: 0.94 vs. 0.90, *p* = 0.034), the coverage was better, and the doses to bladder and maximal dose to rectum were significantly lower in BT plans. The authors conclude that interstitial BT provides better dosimetry compared to IMRT and recommend it as a standard treatment for cervical cancer patients who are not suitable for intracavitary BT. SBRT with CK was also compared to BT. In a feasibility study, Cengiz et al. [29] generated SBRT plans on CT scans of 11 patients treated with tandem and two ovoids. The BT dose distributions were prescribed to point A. In the SBRT plans, they found better dose distributions in the PTV and lower maximum doses to critical organs at the expense of higher doses to the bone marrow. Neumann et al. [30] investigated CK robotic radiosurgery as an alternative to BT for cervical cancer. They calculated BT and CK treatment plans simulating BT-like dose distributions with different homogeneity for 11 patients and compared the plans with each other and with 38 other patients treated with BT. In CK plans with BT-like inhomogeneity, OAR constraints could not be met, but when the maximum dose was limited to 143% for CK, OAR protection was similar to BT, with better target coverage and conformity. They concluded that CK with a moderately inhomogeneous dose distribution is an equivalent option for boost irradiation of cervical cancer patients.

In a recent paper, a dosimetric and radiobiological comparison of IMRT and intracavitary (IC) BT boost following 3D-CRT whole-pelvis irradiation for gynaecological cancer was performed [33]. IMRT boost plans were prepared for 15 patients to mimic IC BT. In terms of conformity and homogeneity, IMRT performed better, but the small volume of OARs received a lower dose using BT. The D_2cm3_ for rectum, bowel and bladder were 47.9%, 39.1% and 30.9% for BT and 89.5%, 91.1% and 78.6% for IMRT, respectively. The NTCP (normal tissue complication probability) values were higher for 3D-CRT plus BT than for 3D-CRT plus IMRT, but it should be noted that BT included three different fractionation schemes with both HDR and PDR treatments.

#### Endometrium

Jones et al. [35] evaluated the feasibility of SBRT dosimetry for early-stage endometrial cancer and compared the dosimetry with HDR-BT. SBRT treatment plans for helical tomotherapy (HT) were generated on CT scans of 10 patients previously treated with IC HDR-BT. Identical OAR contours were used, and PTV in SBRT plans was created by a 2-mm extension of the primary CTV (uterus plus cervix). SBRT achieved significantly higher target volume coverage than BT, but the volume of the uterus irradiated with the 150% isodose was higher with BT (22.7% vs. 16.7%, *p* = 0.05). The dose to the bladder was slightly lower for SBRT but higher to rectum and sigmoid. For bowels and femoral heads, BT resulted in significantly lower doses compared to SBRT. Based on these results, the authors conclude that SBRT with HT is a feasible treatment option and is regarded as a reasonable potential back-up alternative to IC BT for endometrial cancer. In another comparison of HDR-BT and SBRT for inoperable endometrial cancer, doses to normal tissues were higher for SBRT plans [36].

#### Vagina

Dosimetric comparisons for vaginal irradiation have also been published [37–40, 71, 72]. Aydogan et al. [38] analysed the dose distributions of IMRT versus HDR-BT in the vagina during postoperative irradiation of patients with early endometrial cancer. Ten patients treated with BT were selected and IMRT plans were generated on the same CT scans. When the dose was prescribed at a 0.5-cm distance from the surface of the cylinder, IMRT resulted in a lower dose to rectum (D_mean_: 19.6% vs. 33.5%) and bladder (D_mean_: 25.9% vs. 32.5%), with similar target coverage and less inhomogeneity. However, the integral dose was 7.2% higher for IMRT. For most parameters, Yildrim et al. [39] found very similar results with comparative dosimetry between BT, VMAT and HT for 12 patients, except for bladder D_2cm3_, which was significantly lower for BT. Recently, a study on irradiation of the vaginal cuff with VMAT and BT in postoperative endometrial cancer was published [37]. Using BT CT scans of 8 patients, two different VMAT plans were generated, and equivalent uniform doses (EUD) were calculated to account for different dose heterogeneities. Dose distributions were more heterogeneous for BT, with a mean EUD for CTV of 136.9% vs. 130% and 111.0% for BT and the two types of VMAT, respectively. Near-maximal doses for rectum and bladder were significantly higher in the BT plans (D_0.1_ _cm3_: 131% vs. 112% for rectum and 129% vs. 108% for bladder). However, BT was superior in terms of dose falloff outside the CTV, dose to femoral heads and integral body dose. These results are in very good agreement with the findings of Pedicini et al. [40]. In contrast, Grelewicz et al. [72] obtained results different from those published before. In their study, using data of only 4 patients, BT plans showed better OAR (bladder, rectum, sigmoid) sparing compared to IMRT and VMAT plans. However, EBRT plans were normalized to achieve the same mean dose for the target volume as in the BT plans.

### Head and neck

**Fig. 1:**
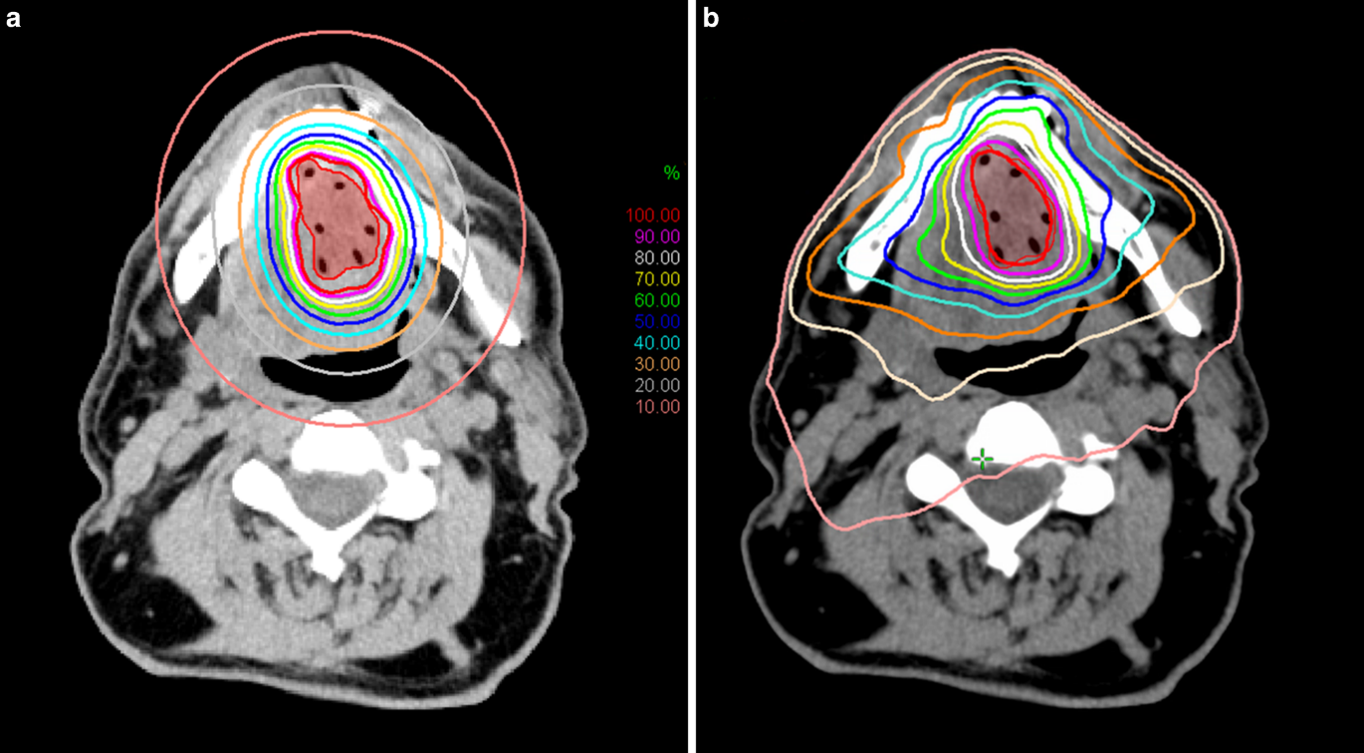
Representative dose distributions for a base of tongue tumour planned with **a** High-dose-rate brachytherapy (HDR-BT) and **b** Volume modulated arc therapy (VMAT)

A comparative dosimetric analysis of target volume and OARs between interstitial HDR-BT and VMAT for localised head and neck cancer was performed at our institute [41]. Thirty-eight patients with tongue, floor of mouth and base of tongue cancer treated with interstitial BT were selected for the analysis. For each patient, a VMAT plan was generated using identical CT data and contours (Fig. 1). The CTV was defined as GTV +5 mm margin limited to mandible. A median of seven catheters (range 3–12) were used in the BT plans and geometrical/graphical optimization was applied without inverse optimization. There was no difference in the target coverage represented by V98 between the two techniques (90.2% vs. 90.4%). In terms of dose to the mandible and spinal cord, BT was the better technique (D_0.1_ _cm3_: 77.0% vs. 85.4%, *p* < 0.05; D_2cm3_: 48.4% vs. 68.4%, *p* < 0.05 for mandible; D_0.1_ _cm3_: 9.7% vs. 12.3%, *p* < 0.05 and D_2cm3_: 9% vs. 10.0%, *p* < 0.05 for spinal cord for BT and VMAT, respectively). Doses to the parotid salivary glands were generally lower for BT. Six out of ten parameters were significantly lower with BT for both ipsilateral and contralateral glands, for example, D_0.1_ _cm3_ (11.2% vs. 18.0%, *p* < 0.05) and D_2cm3_ (7.0% vs. 10.5%, *p* < 0.05) for ipsilateral glands. All parameters for the ipsilateral submandibular glands were lower with BT, but the differences were not significant, whereas for contralateral submandibular glands, all but one parameter was significantly lower with BT. Note that the volumes of the PTVs in this study were exactly the same for both treatment modalities, as there was no additional margin around the CTVs for VMAT planning. However, with EBRT, the target volume is usually larger due to setup errors and patient movement. It follows that if larger PTVs had been used in VMAT plans, the dose to OARs would have been higher, which would have further highlighted the superiority of BT.

### Liver

In one study, virtual BT plans were created on CT images taken for stereotactic ablative radiotherapy (SABR) of liver metastasis, and the BT and SABR plans were compared dosimetrically [42]. PTV was the same for both planning techniques and the comparison was based on the same target volume coverage (V100 = 94%). The mean dose to the small bowel and right kidney was higher for BT (10.8% vs. 7.1% and 6.7% vs. 4.2%), but mean dose to the stomach was similar. For BT, the mean V150 was 63.6%, indicating a significant dose escalation within the PTV. The dose falloff was steeper for BT, but the difference was not significant. It should be noted that only nine comparative plans were analysed, but the authors concluded that BT could result in a dose escalation to the PTV at similar doses to OARs compared to SABR. Another study by Hass et al. [43] included 85 patients with primary or secondary liver malignancy. All patients were treated with interstitial BT using an HDR afterloading technique with an Ir-192 source. A virtual SBRT plan was then calculated for each patient using the original BT planning CTs with catheter placement. To create the SBRT PTV, a margin of 5–10 mm was added to the target volume used in BT, resulting in an increase in the mean PTV volume from 34.7 cm^3^ (BT) to 73.2 cm^3^ (SBRT). The PD was the same (15 Gy or 20 Gy depending on histology), and the same OAR dose constraints were used for both techniques. With BT, the target coverage was better, especially in the 20-Gy PD group. The mean D99.9, which is effectively the minimum dose in the PTV, was 19.9 Gy in BT and 17.5 Gy in SBRT plans (*p* < 0.001). D90 was significantly higher in the BT plans (24.3 Gy vs. 16.5 Gy for 15-Gy PD and 29.2 Gy vs. 20.6 Gy for 20-Gy PD) due to a more heterogeneous dose distribution of BT. For BT, the dose to the liver was lower. In the 20-Gy PD group, the mean V5Gy was 611 cm^3^ in the BT and 694 cm^3^ in the SBRT plans (*p* = 0.001). These volumes correspond to 41.8% and 45.9% of the liver volume (*p* = 0.007), respectively. In the 15-Gy PD group, the same volume parameter was lower in the BT plans, with no statistical difference, but for the whole patient cohort, BT was significantly more favourable. In a recent study, Walter et al. [44] compared 46 HDR-BT treatment plans with virtually planned SBRT plans. Using EQD2 (equivalent dose in 2-Gy fractions) and clinically relevant doses, BT was found to be superior in sparing normal liver tissues. Target coverage was better, but conformity was worse for BT.

### Lung

In one study, Milickovic et al. [16] selected 5 patients with intrathoracic malignancies and compared BT and SBRT treatment plans using identical sets of structures. In the BT plans, the number of catheters ranged between 6 and 10 and an inverse optimization technique was used. In terms of target coverage by the PD, V100 was higher for BT (93% vs. 89%, *p* = 0.03), but there was no difference between the two modalities for V95 and V90. Conformity was better (COIN: 0.84 vs. 0.79, *p* = 0,134) and the dose gradient was significantly higher (R_50_ : 2.47 vs. 4.03, *p* = 0.002) for BT. The maximum spinal cord dose was 12.8% in BT and 21.1% in SBRT plans (*p* = 0.022). Differences in the other dosimetric parameters were not significant for OARs, but BT resulted in lower values for three out of five parameters. Chan et al. [45] performed a dosimetric analysis between HDR-BT and SBRT for primary and metastatic lung lesions. Virtual BT plans were created for 9 patients treated with SBRT using a single catheter. For both techniques, the target volume included the GTV with a 3-mm margin to account for tumour deformation. The mean GTV was 7.2 cm^3^. Since the most advanced technology of real-time tumour tracking was used for SBRT, there was no difference in PTV volumes (no CTV–PTV margin). Two VMAT planning approaches were used, a margin-based and a robust optimization method. Dose distributions were calculated using the same category of dose calculation algorithms (collapsed cone convolution), with tissue heterogeneity corrections also in BT planning [77]. The PD was 34 Gy and the dose constraints for OARs were based on the RTOG protocol 0915 [78]. In the BT plans, the mean GTV and PTV doses were significantly higher (GTV: 122.2 Gy vs. 50.4 Gy and PTV: 65.1 Gy vs. 43.4 Gy, *p* < 0.05, respectively) and the R_50_ dose gradient (volume ratio of 17 Gy to 34 Gy) was better (2.9 vs. 4.3, *p* < 0.05). The results from the margin-based method for SBRT plans are given below. In the low dose range, the whole lung received a higher dose with BT (V_5Gy_: 239 cm^3^ vs. 237.7 cm^3^), but the trend was reversed at higher doses (V_7Gy_: 153 cm^3^ vs. 156 cm^3^ and V_20Gy_: 34.7 cm^3^ vs. 36 cm^3^). Dose constraints per the RTOG 0915 protocol were achieved for all critical organs except in some cases for the chest wall in BT. However, the overall mean D_max_ and D_1cm3_ were lower in the BT plans (22.6 Gy vs. 25.0 Gy and 15.2 Gy vs. 18.4 Gy, respectively). All other DVH metrics showed no statistical difference between BT and SBRT. Based on their results, the authors conclude that SBRT using an advanced MLC tracking technique and non-coplanar VMAT can achieve dosimetric quality comparable to HDR-BT, and that the high GTV dose in BT may be beneficial for enhanced killing of radioresistant tumour cells.

### Prostate

For localized prostate cancer, BT has been the treatment of choice for decades, including LDR seed implantation and HDR monotherapy, as well as for a boost following or during teletherapy. But various EBRT techniques are also available and have excellent results in clinical applications. In an early study, HDR-BT dose distributions for prostate cancer were approximated by CK with very similar dosimetry but using identical PTVs for both techniques [47]. HDR-BT plans were also compared with step-and-shoot IMRT and HT [48]. At the same target coverage (V95 = 100%), the rectum received the lowest dose with BT (D_mean_: 3.9 Gy vs. 6.6 Gy in IMRT and 5.6 Gy in HT). The bladder was also better spared by BT, and V10% for normal tissue was more than eight times lower with BT than with IMRT or HT. In another early study, treatment plans of HDR-BT and IMRT for localised prostate cancer were compared using two separate patient cohorts [50]. In the BT plans, rectum and bladder received lower doses with very similar target coverage. Sudahar et al. [51] investigated the similarity between the dose distributions of CK and HDR-BT. Using equivalent doses, they found similar peripheral target doses between the two treatment techniques, but higher doses within the PTV and a steeper dose gradient in BT plans. Spratt et al. [52] performed a dosimetric analysis with 5 patients, creating virtual SBRT plans and attempting to reproduce the HDR dose distributions. In the SBRT plans, PTVs were created from 100% isodose lines of the BT plans. This resulted in very similar target coverage, but BT was better in most rectal and bladder parameters. Intraprostatic doses were much higher in the BT plans, and these could not be achieved with SBRT even when direct violations of OAR dose constraints were allowed. A group in Vienna performed a dosimetric comparison between BT (LDR, HDR) and EBRT (VMAT, proton, carbon ion) techniques with particular emphasis on the dose to OARs [53]. For each of 10 patients with localized prostate cancer, five individually optimized treatment plans were generated using the same contours. The dose distributions were converted to a radiobiologically identical fractionation. Although EBRT techniques represented the most advanced intensity-modulated treatment options with photons (VMAT), scanned protons (IMPT) and carbon ion (IMIT), BT techniques were clearly superior, especially in terms of dose to the bladder wall, rectal wall and normal tissues, with the lowest values. The D_mean_ for rectal wall was 10.5 Gy for HDR-BT and 28.2 Gy for VMAT, and in between for the other techniques. The V_50_ in cm^3^ was 1.5, 4.1, 7.2, 7.0 and 4.7 for HDR-BT, LDR-BT, VMAT, IMPT and IMIT, respectively. In the same order, the V_50_ for bladder wall was 0.6, 2.8, 5.5, 5.3 and 3.2 cm^3^, respectively. For the five techniques, the volumes of normal tissues that received 30% of the PD were 42, 120, 487, 198 and 253 cm^3^. The D_mean_ for urethra was lowest for HDR-BT (62.9 Gy) and highest with LDR-BT (106.6 Gy). The BT techniques resulted in negligible doses to femoral heads (D_mean_ < 2 Gy), while the EBRT techniques resulted in a D_mean_ of nearly 20 Gy on both sides. In another study by Fukuda et al. [44], when HDR-BT monotherapy plans were simulated with SBRT using common CT images and contour datasets (except PTV), SBRT was found to be significantly superior in almost all dosimetric parameters of the bladder and urethra. In the BT plans, however, rectum was significantly better spared.

Dosimetry of boosting of the dominant intraprostatic lesions (DIL) with VMAT, intensity-modulated proton therapy (IMPT) and HDR-BT was investigated by Andrzejewski et al. [55], and HDR-BT was found to be superior to VMAT and IMPT in terms of OAR sparing and DIL boosting. For example, the equivalent uniform dose (EUD) was 49.4 Gy, 47.9 Gy and 28.1 Gy for bladder, 53.3 Gy, 50.5 Gy and 35.1 Gy for rectum and 88.3 Gy, 86.6 Gy and 62.4 Gy for urethra in the order of VMAT, IMPT and HDR-BT, respectively. In a study by Eade et al. [57], stereotactic radiation therapy boost for prostate cancer was used to mimic the HDR-BT boost, and the SBRT dose distribution was found to be similar to BT. For the intraprostatic urethra, D10 was higher, but for bladder and rectum, the D_1cm3_ was lower with BT. Sannamed et al. [58] used single-fraction HDR-BT or integrated VMAT boost for tumour-targeted dose escalation in localized prostate cancer, treating 40 patients with both techniques. Dosimetry was compared using equivalent dose levels in 2‑Gy fractions (EQD2). Doses to OARs were comparable, but both rectal and bladder walls and urethra received lower doses with BT. Furthermore, mean and maximal target doses were higher with BT (*p* < 0.05). Due to its dosimetric characteristics, the authors consider HDR boost as the best technique to deliver boost dose to the GTV located near critical structures. In a recent study, Chatzikonstantinou et al. [59] compared CK SBRT plans with virtual HDR-BT plans based on the same 15 patients. The BT plans were ultrasound based and the PTV was equal to the prostate gland without margins. For a direct comparison of DVH values, a hypothetical single-fraction 35 Gy was used in the BT plans. In the SBRT plans, anisotropic margins of 3–5 mm around the GTV were used to create the PTV and a dose of 35 Gy was delivered in five fractions. Dose coverage of the GTV by the PD was higher in the SBRT plans (99.98% vs. 90.07%), but intraprostatic high-dose volumes were larger in BT the plans. The maximum rectal and bladder doses were significantly lower with BT (30.3 Gy vs. 36.4 Gy, *p* < 0.0001 and 32.7 Gy vs. 37.4 Gy, *p* = 0.0004, respectively). However, in terms of urethral dose, HDR-BT was worse, with a maximum dose of 45.6 Gy compared to 40.8 Gy (*p* < 0.0001) for SBRT. From these results, the authors conclude the superiority of HDR-BT for rectal and bladder dosimetry. Fröhlich et al. [60] performed a comparison of two BT (LDR and HDR) and two EBRT (VMAT, CK) techniques using the CT images sets of 10 patients treated with CK. EQD2 doses were calculated and compared. The EBRT plans were more conformal than the BT plans, with D_2cm3_ for the rectum and bladder being lowest in HDR-BT (36 Gy and 51.4 Gy), while D_0.1_ _cm3_ for the urethra was lowest (79.9 Gy) in VMAT. The hips and sigmoid were best protected by HDR-BT. In a feasibility study, Willigenburg et al. [61] created comparative SBRT treatment plans on an MR-linac system for 30 patients treated with focal salvage HDR-BT. Since intrafractional prostate motion detection and online replanning is available with the MR-linac, only a 1-mm CTV–PTV margin was used in the SBRT plans. The target coverage was very similar, but the urethra and rectum received a significantly lower dose with BT. At a PD of 19 Gy, the median D10% was 16.0 Gy vs. 17.5 Gy (*p* < 0.001) for the urethra, and D_2cm3_ was 8.6 Gy vs. 10.7 Gy (*p* < 0.001) for the rectum in the BT and SBRT plans, respectively.

### Skin

Six studies have been published on the dosimetric comparison of surface mould HDR-BT and VMAT for the treatment of skin lesions on the scalp and the extremities [62–67]. Boman et al. [63] used four and three plans of patients treated with HDR-BT surface mould on the scalp and lower leg, respectively, and retrospectively created VMAT plans with the PTV created on the CTV of BT plans with an additional 3‑mm setup margin. For scalp cases, the conformity was always better for VMAT, D95 for CTV was similar and the mean CTV dose was higher for BT. The mean dose to brain was higher with BT and D_0.1_ _cm3_ was very similar. Similar observations were found for the leg cases, except for the D_0.1_ _cm3_ of normal tissue, which was lower for BT. In a recent study, Wills et al. [66] compared the plan quality of HDR-BT and VMAT for superficial treatment of skin on the scalp and lower limbs with significant curvature. BT plans were retrospectively re-planned for VMAT using identical CT data and CTV/OARs. The conformity index was significantly higher for VMAT. The CTV and PTV coverage by 95% of PD was similar in all cases. In scalp cases, the optic system and the lenses generally received a lower dose in VMAT, similarly to the brain. With BT, small volumes of normal tissue received high doses were similar to or higher than with VMAT, and the maximum dose to the skin surface was higher with BT. Using a relatively large cohort of 37 patients, Buzurovic et al. [67] dosimetrically compared HDR-BT and electron irradiation used for skin lesions of different sizes in various body sites. The quality of the plans was highly dependent on the geometry of the target, including size, curvature and topology. For the two techniques, target D_2cm3_ values were comparable, but doses to smaller volumes (0.5 cm^3^, 0.1 cm^3^) were higher with BT. For complex targets, BT provided excellent target coverage, as the dose could be adjusted to irregular surfaces using flexible flap applicators. There were complex cases where only BT provided clinically acceptable plans. For smaller, flat targets, electron beams provided plans similar to BT, with better dose homogeneity.

### Dose calculation algorithms

The dose calculation algorithm is not always reported in publications. In most cases, only the name of the planning system is mentioned, especially for EBRT. In ten studies, neither the calculation algorithm nor the planning system was given. In nine cases the algorithm (collapsed cone, AAA, Monte Carlo) and in the other cases only the name of the planning system was given. As for BT, the TG-43 formalism was used in all but six studies. In four papers, no information on the method of dose calculation was provided and more advanced model-based dose calculation algorithms (MBDCA) were used only in two studies [45, 63]. Chan et al. [45] calculated BT dose distributions in the lung using the Advanced Collapsed Cone Engine (ACE; Elekta Brachytherapy, Veenendaal, the Netherlands). Boman et al. [63] used the Acuros BV (Varian, Palo Alto, CA, USA) and the TG-43 algorithm for dosimetric calculation in surface mould HDR-BT. They compared the dosimetric parameters calculated by both algorithms. CTV coverage (D95) was decreased by more than 13% for scalp cases and by at least 5.8% for lower leg cases when Acuros BV was compared with TG-43 plans. A 1-cm bolus (as a backscatter material) reduced the difference by 3% and 2% for the scalp and lower leg cases, respectively. Near maximum doses were higher for TG-43 (by 9.1% for scalp and by 4.8% for lower leg).

## Discussion

**Fig. 2:**
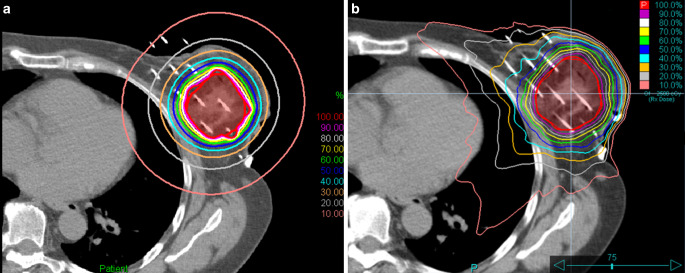
Representative dose distributions for partial breast irradiation planned with **a** HDR multicatheter interstitial BT and **b** stereotactic irradiation with CyberKnife (Accuray Inc, Sunnyvale, CA, USA)

In HDR-BT, different irradiation times can be used in different source positions using the stepping-source technique. Because of this feature, this technique can also be called intensity-modulated brachytherapy (IMBT) [79]. In modern EBRT, such as stereotactic radiosurgery (SRS) or SBRT, small fields or small beam segments are usually used for generating intensity modulation, where the dose gradient perpendicular to the beam direction is very steep at the beam edges. Along the main axis of the beam, however, the dose does not vary rapidly with distance, and is characterized by the slope of the depth dose curve, which is determined primarily by the energy. In a real treatment plan, when multiple beams (3D-CRT or IMRT) or arcs (VMAT) are used, the dose gradient in any direction is a combination of the two very different dose gradients mentioned above, and their weightings determine the final dose gradient. With proper beam arrangement and weighting, the dose to an OAR located in any direction relative to the target volume can be kept low, but only at the cost of a higher dose delivered in the other direction. With this flexibility, the shape of the dose distribution can be changed almost arbitrarily. In BT, this directional dependence is absent. The dose distribution around a source is nearly uniform, with only a slight anisotropy along and near the source axis, and cannot be spatially modulated. However, with the right geometric arrangement of multiple sources, the dose distribution can be tailored to any shape. At a distance of a few centimetres, however, the isodose curves will be nearly circular with no directional dependence (Figs. 1 and 2). This means that the dose to an OAR at a greater distance can only be reduced by decreasing the PD and compromising the target coverage. However, the doses to OARs close to the target volume can be kept low due to the large effect of the inverse square law. This claim is supported by the results of some studies showing that skin, ribs and small ipsilateral lung volumes exposed to high doses are better protected by BT than by EBRT [20, 22, 23].

Currently, the TG-43 protocol is the standard calculation formalism in clinical BT planning systems and was the most common calculation method in the cited studies. The vast clinical experience gained in recent decades is based on this method. It is important to note, however, that the MBDCAs using radiation transport in heterogeneous medium are already available for BT, but their application is still mainly limited to the field of research [77]. The results of the TG-43 and MBDCA algorithms are very similar in standard cases, but differences more than 10% can occur in extreme geometries [63].

Comparative dosimetric studies of BT and EBRT fall into two categories: either they use two separate groups of patients, or the planning is performed using the same CT image set and the same contours (Table 1). In the latter case, either a BT or an EBRT CT image set is used for reference dosimetry. If two separate patient cohorts are chosen for comparison, some inconsistencies between contours may exist. In this case, however, real clinical treatment plans are compared without virtual simulation. Studies based on identical CT image sets and contours exclude interobserver variations in target definition and delineation, but one plan is always virtual, which is never realized. When BT plans are created based on the data used for EBRT treatments, the virtual BT plans represent an ideal applicator/catheter geometry that is never actually feasible. In real plans, the catheters are never parallel, and the target volume cannot always be geometrically covered by the catheters. So, the quality of the virtual plan is always better than what can be achieved in real cases. When the BT plan is chosen as a reference, the patient’s anatomy and position are not the same as those used for EBRT. Furthermore, the presence of applicators/catheters in CT images causes heterogeneity in mass density and this affects the dose distribution of EBRT.

One of the advantages of BT is that due to the absence of a CTV–PTV margin (PTV equals CTV), the target volume to be irradiated is smaller. This fact probably contributes to the favourable dosimetric characteristics of BT, in particular to the improved OAR protection. In EBRT plans, depending on the treatment site and the irradiation technique, with or without motion management, PTV is generally larger than CTV. Table 1 shows the CTV–PTV margins used in the EBRT plans. In most cases, no specific motion management was applied, but an additional margin was added to the CTV to account for organ motion and setup inaccuracies. This margin varies between 2 and 10 mm. However, there are studies in which the same target volume was used for BT and EBRT techniques [27, 29, 30, 32, 41, 42]. It follows that the benefits of BT were underestimated in these studies and that the dosimetric values would have been more favourable towards BT if a larger volume (CTV + margin) had been used for EBRT planning. In the CK plans with the synchrony tracking system, the CTV–PTV margin varied between 0 and 5 mm. MLC tracking on conventional linear accelerators was used in three studies [45, 57, 61]. A margin of 3 mm was added to the CTV for lung and 7 mm for prostate [45, 57]. Using an MR-linac with an online re-planning feature, only a 1‑mm margin around the CTV was applied [61].

In cervical cancer, intracavitary BT is mandatory, as confirmed not only by dosimetry but also by clinical results [12, 13]. In other studies, IMRT and SBRT were used to mimic BT dose distribution, and it was investigated whether EBRT can replace or be used as an alternative to BT [35, 38, 73].

Interestingly, for irradiation of the vaginal cuff, EBRT techniques performed better than BT in terms of dose to OARs [37–40]. This is probably due to the fact that in BT, the dose is prescribed relatively far from the HDR source (2.5–3.5 cm) and the inverse square law is no longer of much advantage at this distance, especially considering that the OARs (rectum, bladder) are very close to the target volume. The advantages of BT include a higher mean dose in the PTV due to a more heterogeneous dose distribution, and a lower body integral dose. Furthermore, it should be noted that the comparative dosimetry in all studies was based on CT scans taken for BT, and that a vaginal cylinder was in place. The EBRT plans were virtual, but a similar applicator should be used as an immobilization device for real treatments, which can also be used for applicator-guided irradiation.

SBRT is the standard technique for ablative treatment of liver cancer. However, with interstitial BT, a more favourable dose distribution can be achieved, with better target coverage and a lower liver dose [42]. The better dosimetry is partly due to the use of additional margins (5–10 mm) in SBRT plans due to respiratory organ motion, resulting in larger target volumes. Lung cancer patients are also good candidates for SBRT. However, for small target volumes, using BT with only a single catheter can achieve a dose distribution similar to MLC-tracking VMAT, with the advantage of a higher dose inside the tumour [45]. For larger tumours, more catheters should be used, which gives the opportunity to create more conformal dose distributions. In terms of target coverage, BT is at least as good as SBRT, with similar conformity and a lower dose to healthy tissues, as the dose gradient is steeper at the edge of the target volume [68]. In partial breast radiotherapy, interstitial BT protects OARs adjacent or near to the target volume better than EBRT, and depending on the location of the target volume, the dose to heart can be kept lower with EBRT. The majority of studies on prostate showed superiority of BT over EBRT for rectal and bladder dose. The skin was the only site where the VMAT plans were better for almost all parameters [62, 63, 65, 66, 70]. The thin, large-curvature target volume appears to be better irradiated by external beams. The reason for poorer BT parameters is that the sources are always outside the target volume, and many of them are located on the opposite side of the PTV and irradiate as “opposing beams”.

A systematic literature review has shown that from a dosimetric point of view, BT can compete with even the most advanced EBRT techniques, in particular with a higher dose centrally within the target volume and sparing of adjacent OARs. The advantage is particularly pronounced for interstitial BT, where adequate geometrical target coverage can be achieved with a large number of needle/source dwell positions, allowing for a conformal and relatively homogeneous dose distribution. If the source positions are outside the PTV (intracavitary gynaecological, surface mould), due to the larger distance, the inverse square law is not as effective as in interstitial cases.

The recent decline in BT can be attributed to a number of factors [3], including the increasing technological sophistication of EBRT, unfavourable reimbursement policies, declining interest in training among residents and a lack of experience of trainees [80, 81]. Inadequate communication with other professionals, lack of media campaign and access to social media may also contribute to less use of BT [82, 83]. To make BT better known to professionals, practical BT education needs to be developed using innovative methods [84, 85], and to complement formal trainings of practitioners, websites providing peer-to-peer medical and educational information (https://www.brachyacademy.com) as well as different multimedia and social media platforms for patients can be used [82, 83, 86].

A limitation of this work is that only a dosimetric comparison between BT and modern EBRT techniques was performed, without examining clinical outcomes. Our intention, however, was to evaluate only the physical characteristics of BT against emerging external beam radiation techniques, including IMRT, VMAT and CK-based robotic radiotherapy. The strength is a comprehensive literature review of the different treatment sites where a planning comparison between BT and EBRT had been published, and our final conclusions on the role of BT were based on these.

The history of BT has been constantly accompanied by technological developments, and further advances are expected in the future in imaging, dose calculation algorithms, physical and biological dose optimization, new applicators, electromagnetic tracking, 3D printing and new isotopes [87–89]. We firmly believe that image-guided BT, supported by inverse dose planning, will compete successfully with high-tech EBRT in the coming times, and we sincerely hope that BT will survive for a long time to come, and serve as a valuable modality for cancer treatment.
